# Supplementary material for: Seeing structural evolution of organic molecular nano-crystallites using 4D scanning confocal electron diffraction (4D-SCED)
Source: Nat Commun. 2022 May 25;13:2911. doi: 10.1038/s41467-022-30413-5 (PMC9132979; doi:10.1038/s41467-022-30413-5)
Supplement: Supplementary file 1 — Supplementary info [file 41467_2022_30413_MOESM1_ESM.pdf]

SUPPLEMENTARY INFORMATION

Seeing Structural Evolution of Organic Molecular Nano-crystallites Using 4D Scanning Confocal Electron Diffraction

Mingjian Wu[1], Christina Harreiss[1], Colin Ophus[2], Manuel Johnson[3], Rainer H. Fink[3] and Erdmann Spiecker[1]

[1] Institute of Micro- and Nanostructure Research (IMN) & Center for Nanoanalysis and Electron Microscopy (CENEM), Department of Materials Science, Friedrich-Alexander-Universität Erlangen-Nürnberg (FAU), Cauerstraße 3, D-91058 Erlangen, Germany

[2] National Center for Electron Microscopy, Molecular Foundry, Lawrence Berkeley National Laboratory, 1 Cyclotron Road, Berkeley, CA, USA

[3] Department of Chemistry und Pharmacy, Friedrich-Alexander-Universität Erlangen-Nürnberg (FAU), Egerlandstr. 3, 91058 Erlangen, Germany.

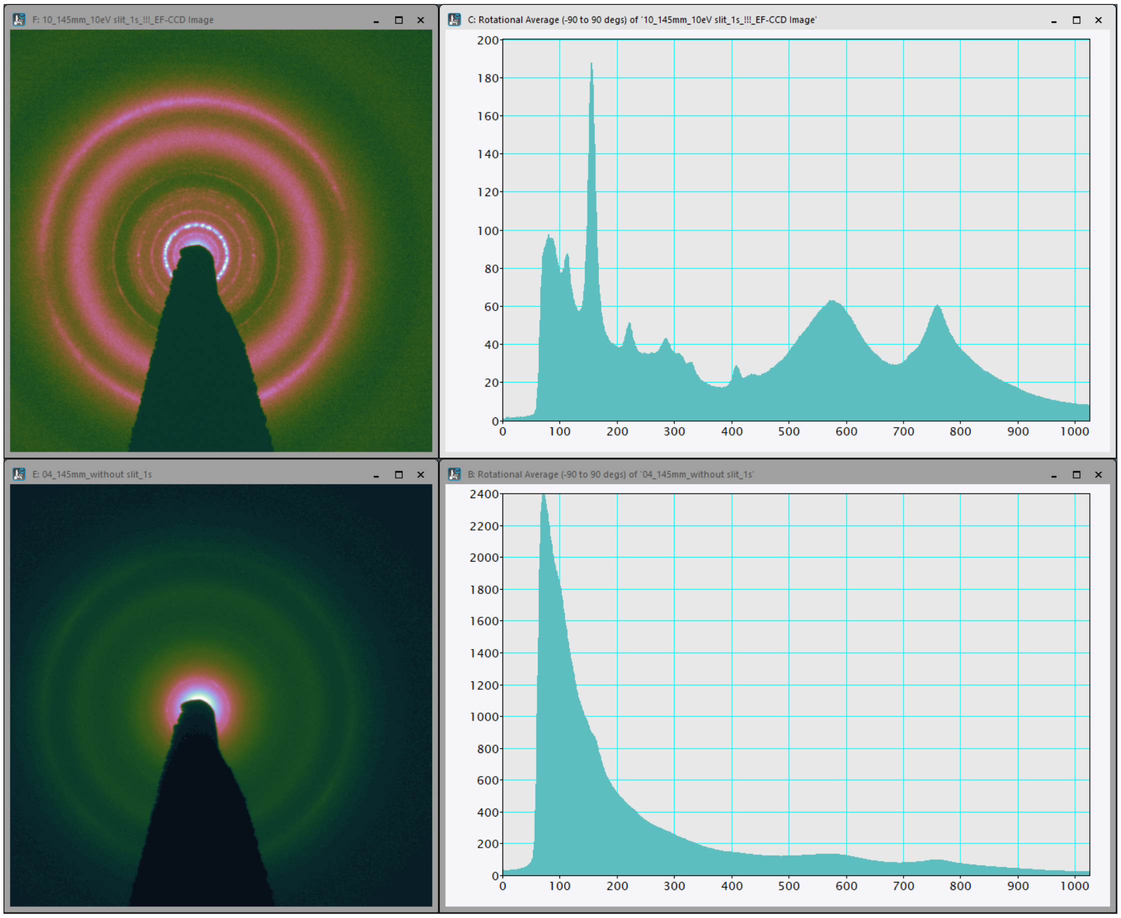

Figure S1. Selection area electron diffraction (SAED) pattern of a ~80 nm thin film of DRCN5T:$PC_{71}BM$ blends after SVA in CS2 for 840s, with (above) and without (below) filtering the elastically scattered electrons using a 10eV energy selection slit around the zero-loss peak. Azimuth integrated intensity profiles are show to the right side. These diffraction patterns are taken from fresh area of the same sample at total dose $<1e^-/A^2$. We note that the enhancement of diffraction peaks to background is dramatic in SAED, but less obvious in NBD and SCED experiments due to the large difference in the ratio of crystalline to non-crystalline substance within the illumination circle, ~3.5 µm in SAED and ~5 nm in NBD and SCED.

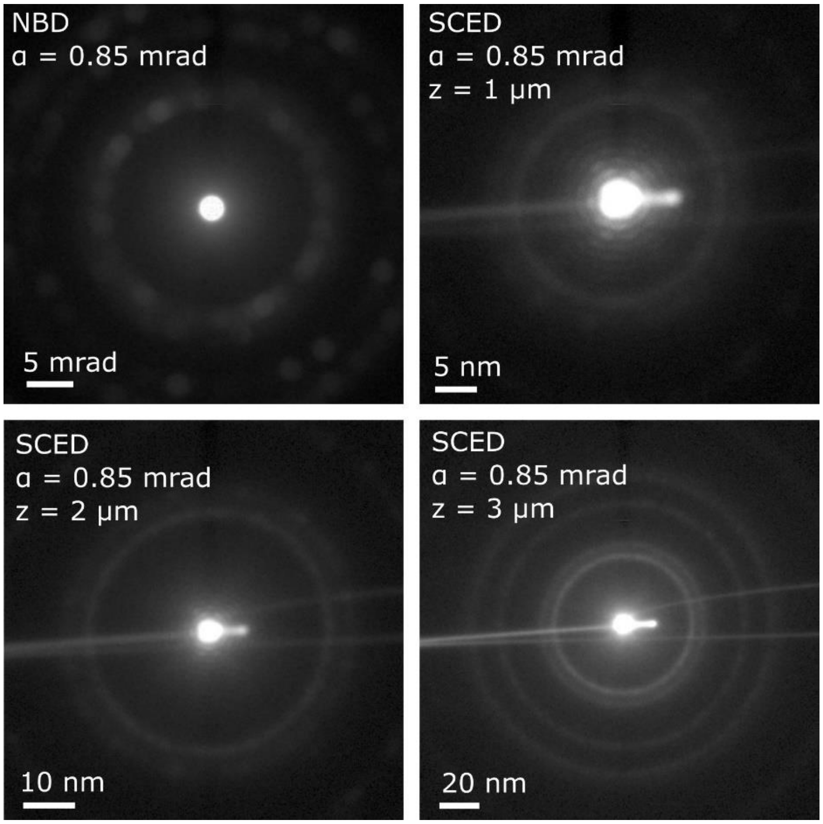

Figure S2. Position averaged (scanning over 450x450 nm area) diffraction patterns of cross-grating (Au nano particles) sample using NBD and SCED setup with different defocus values. The {111} and {002} is hardly distinguishable in NBD but clearly separable in SCED even with small defocus of 1 μm. The Airy disk appear around the center beam is well separated from diffraction spots when defocus z>2 μm.

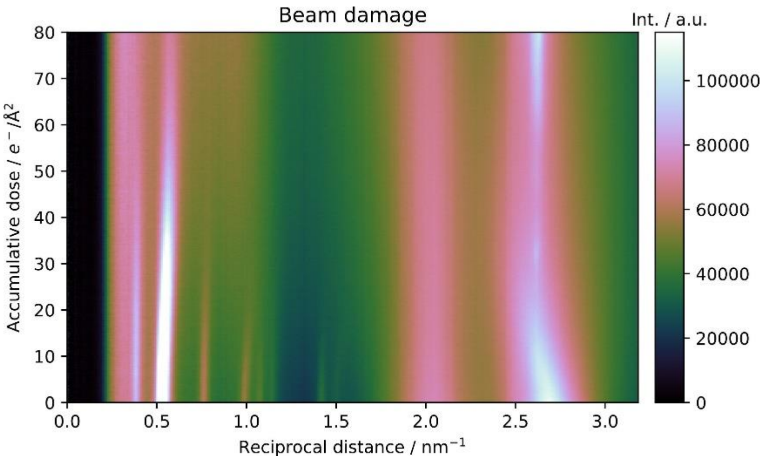

Figure S3. azimuthal integrated diffraction profile as function of accumulated dose, measured at liquid nitrogen temperature.

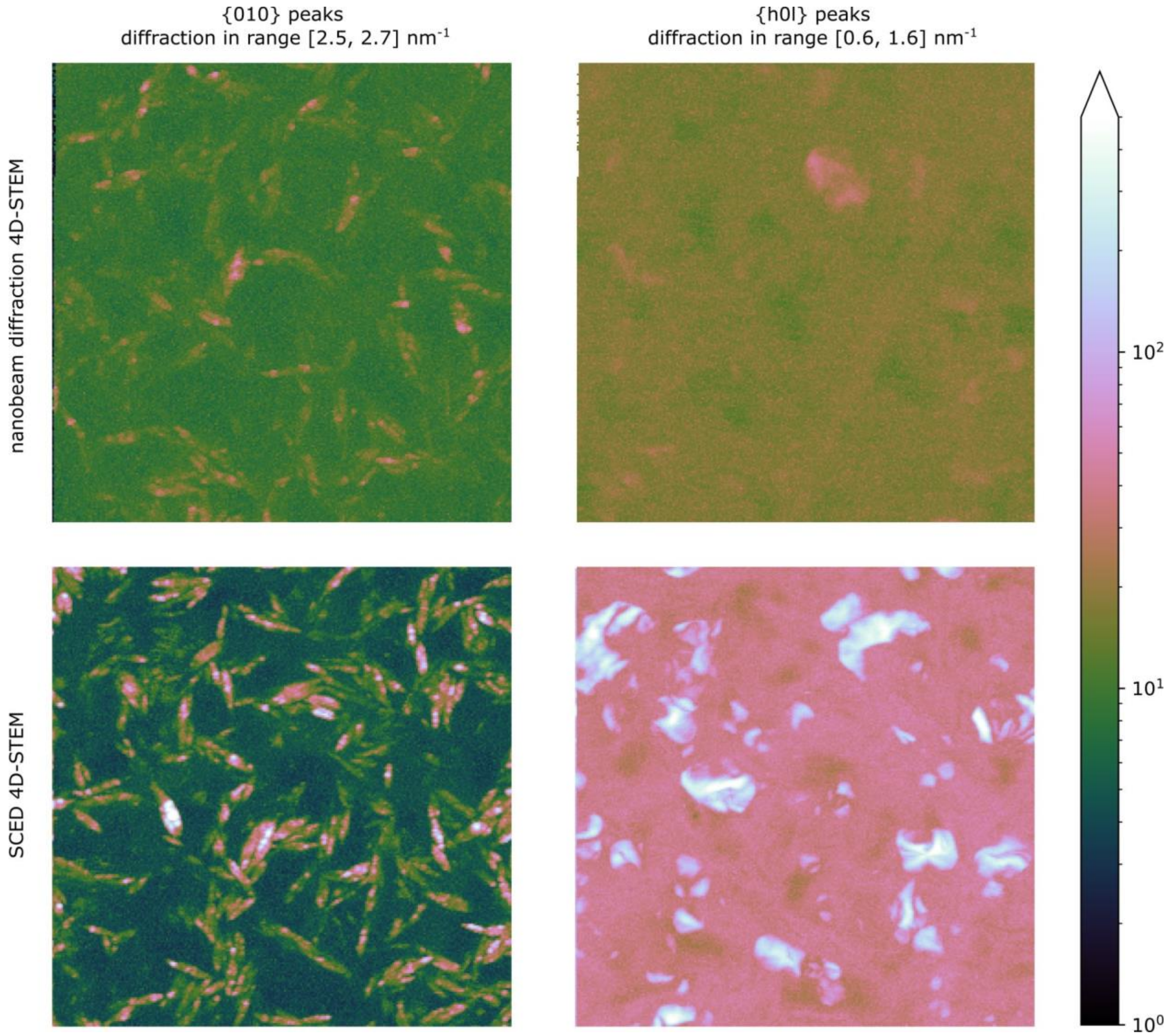

Figure S4. Comparison of the diffraction SNR using NBD setup (upper panels) and SCED setup (lower panels).

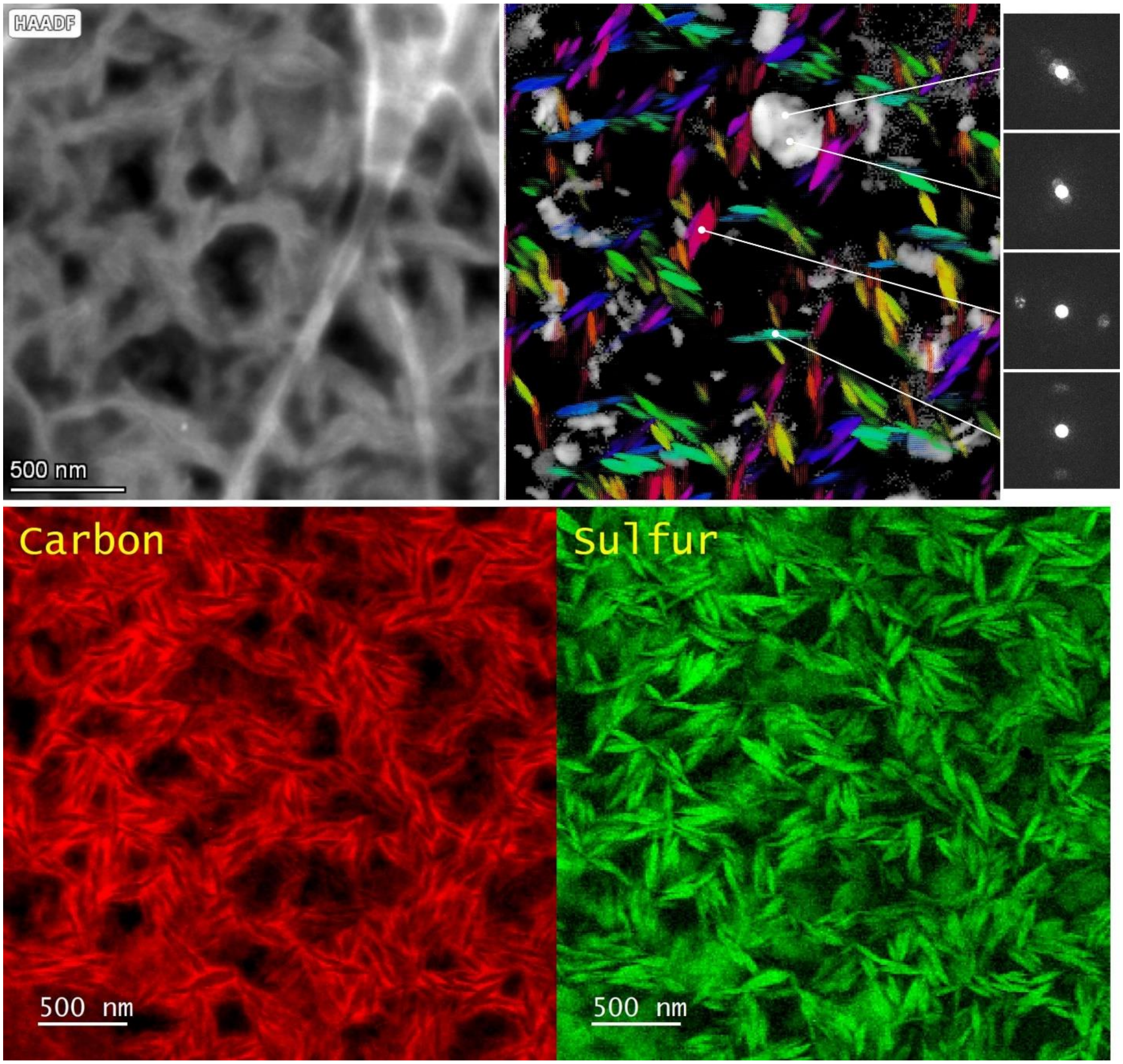

Figure S5. (top-left) STEM-ADF (acquired at the end) of the areas where NBD 4D-STEM was acquired. (top-right) visualization of the edge-on and face-on crystalline domain based on the NBD dataset and some raw data shown as insets. Beam disk overlap makes analysis of orientations of face-on domains difficult. (bottom) carbon and sulfur elemental maps evaluated from STEM-EELS datasets. We note that the electron dose used to obtain the STEM-EELS signals is 3-4 orders of magnitude higher than that applied for diffraction imaging experiments. Sample: thin film blend of DRCN5T:$PC7_1BM$ that has been SVA processed in CS2 for 120s, the same as in Fig. 3b

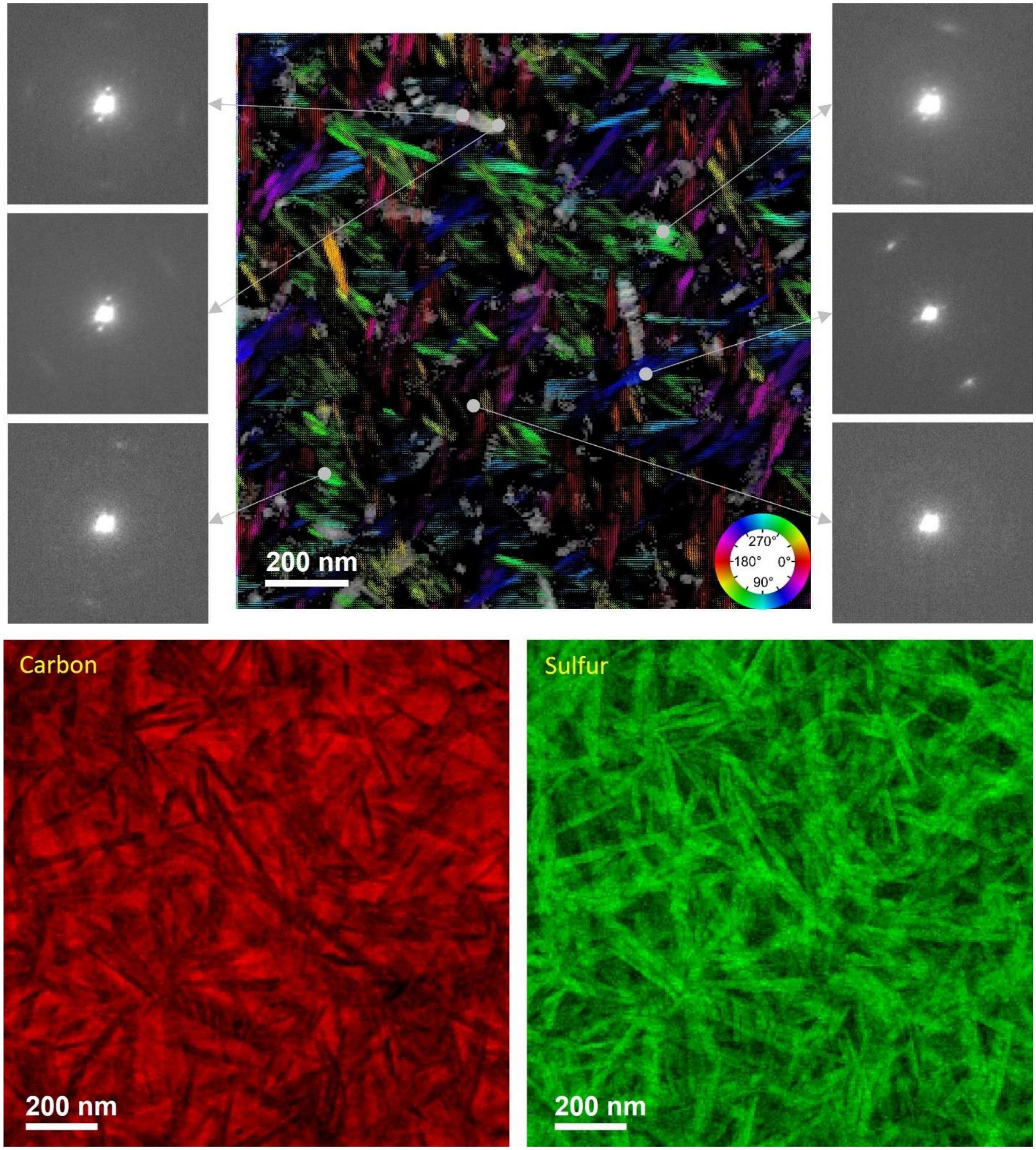

Figure S6. (top) Visualized edge-on (color-wheel representation) and face-on (grayscale) crystalline domains of DRCN5T after the thin film blend of DRCN5T:PC7$_1$BM has been SVA processed in CHCl3 for 120s. Few raw diffraction data is show as insets aside. (bottom) carbon and sulfur elemental maps evaluated from STEM-EELS datasets acquired from the same sample. We note that the electron dose used to obtain the STEM-EELS signals is 3-4 orders of magnitude higher than that applied for diffraction imaging experiments.

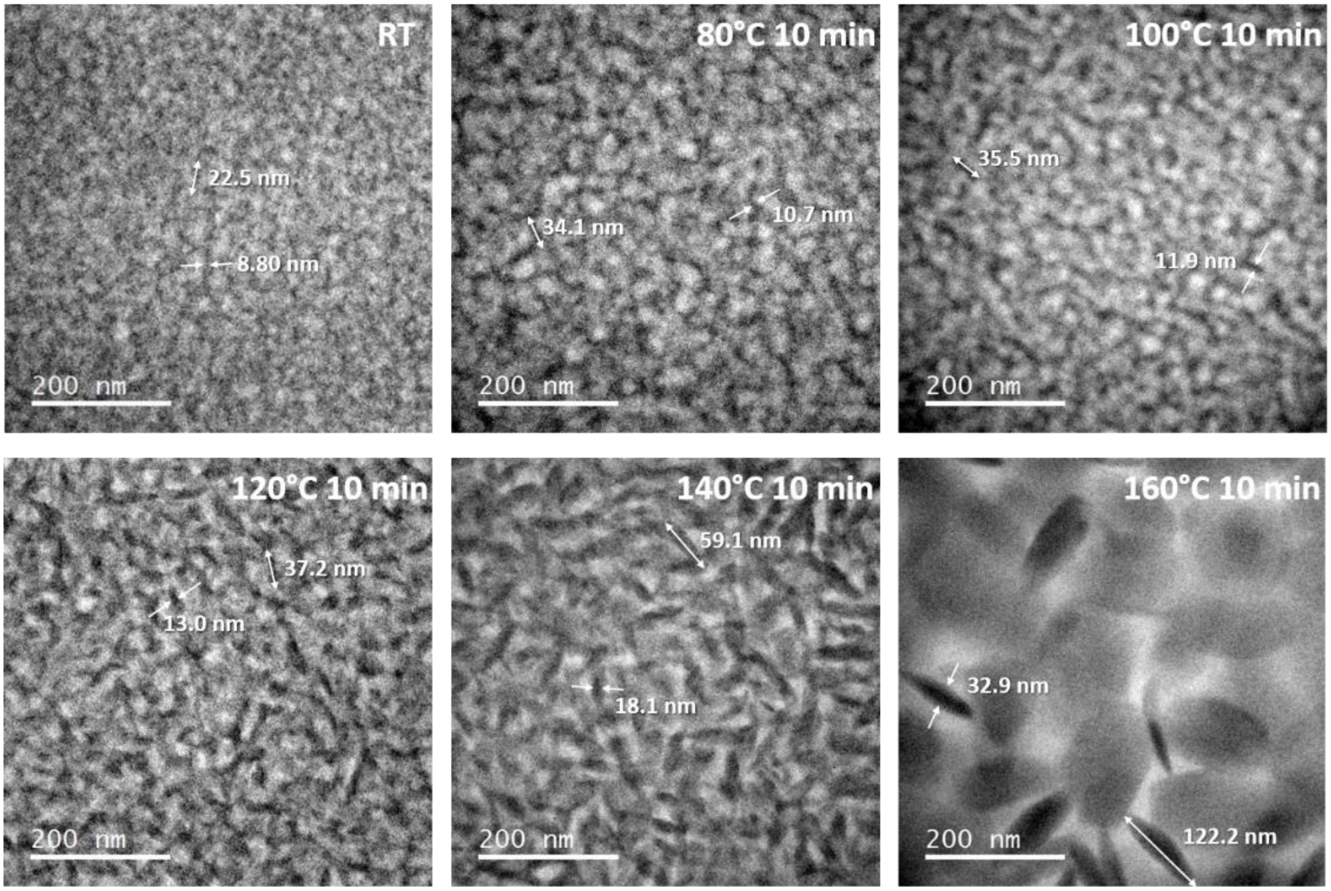

Figure S7. Nanomorphology of the DRCN5T:$PC_{71}BM$ thin film samples after *ex-situ* thermal annealing processes at different temperatures as revealed by carbon elemental maps with EFTEM. Every image represent the morphology of a sample annealed at the indicated constant temperature and time without temperature history (as in the in situ case in Fig. 5).

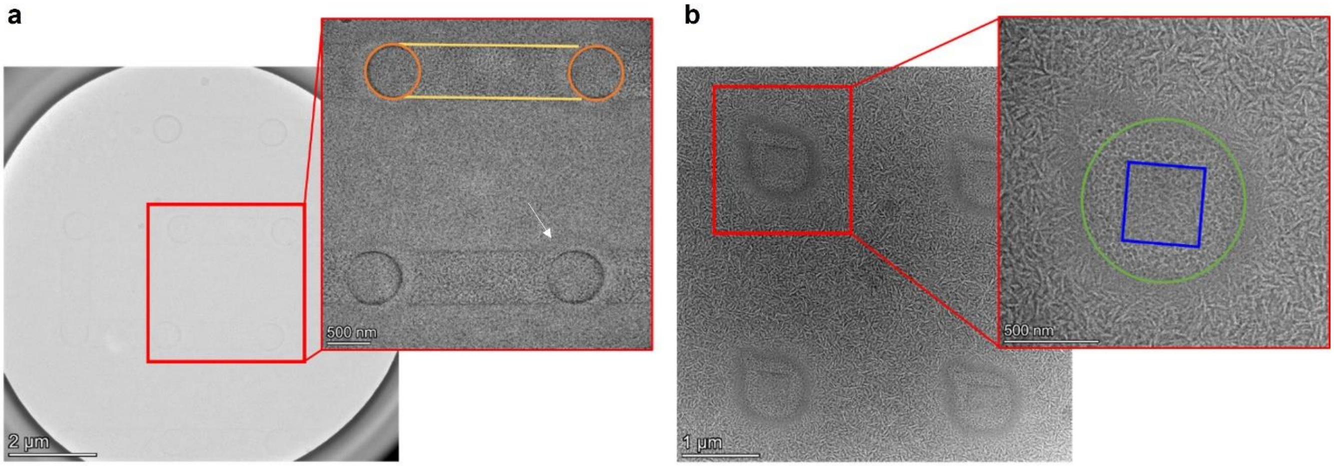

Figure S8. Low-mag image showing the nanomorphology of DRCN5T:PCBM after *in situ* annealing experiments in the TEM. (a) after *in situ* EFTEM observation using parallel illumination. Orange circles indicate the size of illumination. The yellow bands indicate the influenced trace of structures while the sample region is moved from left to right with the beam illumination, the white arrow indicate the delocalized zone of beam influence. (b) after *in situ* STEM-EELS observation. The blue box indicates region of data acquisition. Green circle indicate the delocalized region of beam influence. The structural evolution was frozen by the beam illumination upon first light, and further evolution took place which is obvious comparing it with the surrounding regions.

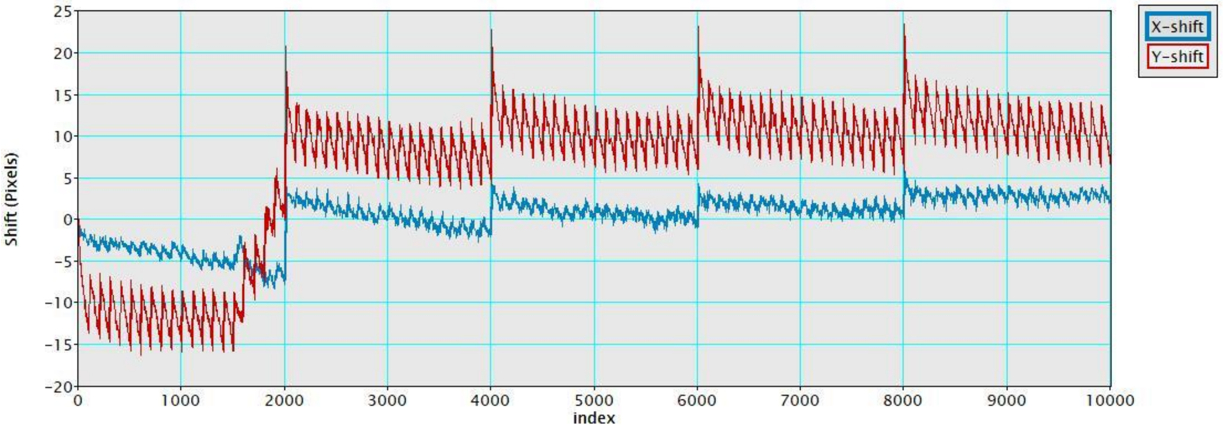

Figure S9. Measured center beam shift as the probe scanning over a field of view of ~1.5 μm. the shift between frames 1500 and 2000 were due to manual shift of the beam to the center of camera.

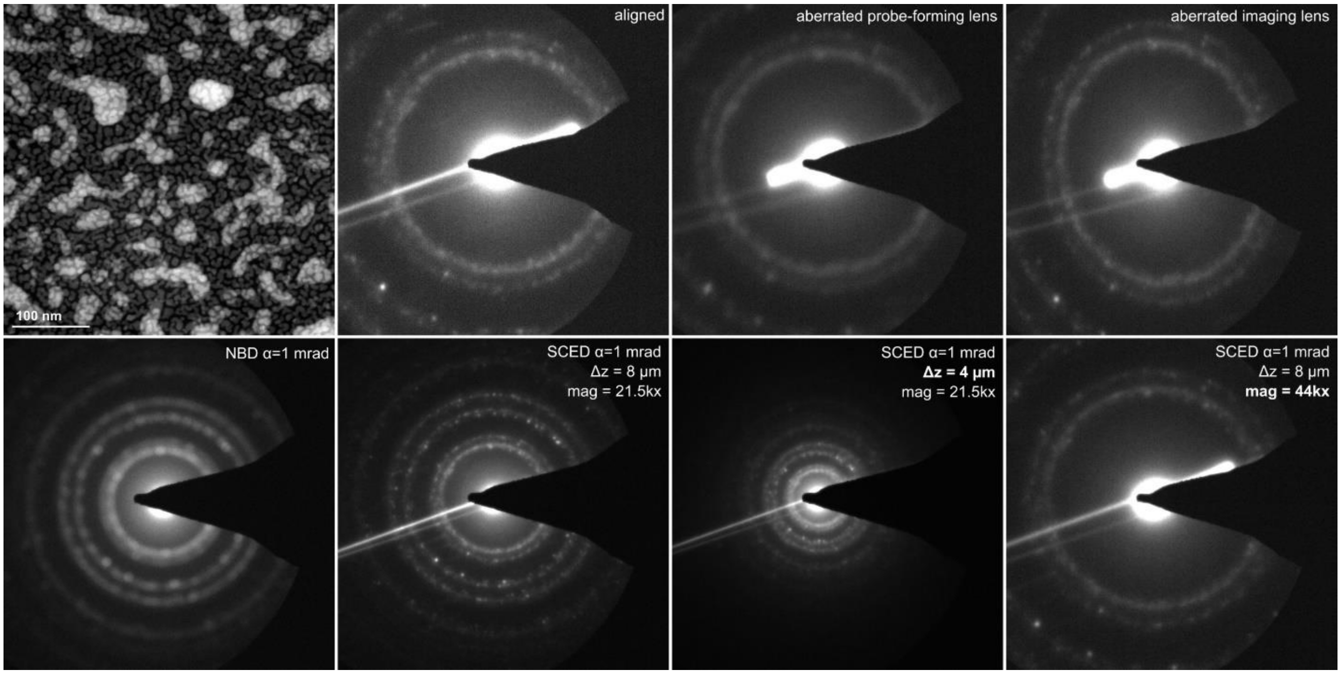

Figure S10. Parameters that dominate the aberration/distortions of SCED patterns demonstrated using a bi-metallic nanoparticle sample. The sample is sputter deposited Au and Pt on each side of SiN_x membrane and dewetted at 600C for 1h. All diffraction patterns are position averaged diffraction patterns, acquired with camera exposure time of 2s while the beam is scanning over a ~1x1 μm region at ~4 fps.
